# Pungency related gene network in *Allium sativum* L., response to sulfur treatments

**DOI:** 10.1186/s12863-024-01206-0

**Published:** 2024-03-26

**Authors:** Ali Ammarellou

**Affiliations:** Department of Biotechnology, Research Institute of Modern Biological Techniques, University of Zanjan, Zanjan, Iran

**Keywords:** Garlic, Pungency, Gene network analysis, Sulfur

## Abstract

Pungency of garlic (*Allium sativum* L.) is generated from breakdown of the alk(en)yl cysteine sulphoxide (CSO), alliin and its subsequent breakdown to allicin under the activity of alliinase (*All*). Based on recent evidence, two other important genes including Sulfite reductase (*SiR*) and Superoxide dismutase (*SOD*) are thought to be related to sulfur metabolism. These three gene functions are in sulfate assimilation pathway. However, whether it is involved in stress response in crops is largely unknown. In this research, the order and priority of simultaneous expression of three genes including *All, SiR* and *SOD* were measured on some garlic ecotypes of Iran, collected from Zanjan, Hamedan and Gilan, provinces under sulfur concentrations (0, 6, 12, 24 and 60 g/ per experimental unit: pot) using real-time quantitative PCR (RT-qPCR) analysis. For understanding the network interactions between studied genes and other related genes, in silico gene network analysis was constructed to investigate various mechanisms underlying stimulation of *A. sativum* L. to cope with imposed sulfur. Complicated network including TF-TF, miRNA-TF, and miRNA-TF-gene, was split into sub-networks to have a deeper insight. Analysis of q-RT-PCR data revealed the highest expression in *All* and *SiR* genes respectively. To distinguish and select significant pathways in sulfur metabolism, RESNET Plant database of Pathway Studio software v.10 (Elsevier), and other relative data such as chemical reactions, TFs, miRNAs, enzymes, and small molecules were extracted. Complex sub-network exhibited plenty of routes between stress response and sulfate assimilation pathway. Even though *Alliinase* did not display any connectivity with other stress response genes, it showed binding relation with lectin functional class, as a result of which connected to leucine zipper, exocellulase, peroxidase and ARF functional class indirectly. Integration network of these genes revealed their involvement in various biological processes such as, RNA splicing, stress response, gene silencing by miRNAs, and epigenetic. The findings of this research can be used to extend further research on the garlic metabolic engineering, garlic stress related genes, and also reducing or enhancing the activity of the responsible genes for garlic pungency for health benefits and industry demands.

## Introduction

In biological ecosystems, along with numerous growth-limiting factors, abiotic and biotic stresses are important challenging physiological barriers that have major effects on plant growth and development. Faced with adverse environmental conditions, plants reprogram their cellular activities through several genes and minimize stress damage using regulatory mechanisms, including post-transcriptional regulation of gene expression [1]. Transcription Factors (TFs) and non-coding RNAs are the important regulatory elements in functional genomics [2]. All biological processes in the organisms are managed primarily by changes in the activity and expression of key genes. The ability of a cell to switch on and off a gene drives all biological function and activity. Because of key effect of genes on metabolism, researchers have focused much interest on gene expression profiling to identify those key genes and gene clusters whose expression changes and variation in different biological states [3–6]. Research studies have shown that changes in gene expression are the key basis of response to environmental factors, stresses, defense response against pathogens, regulation of metabolic pathways, regulation of photosynthesis or symbiosis, so plant by using this possibility and resourcefulness, regulates and manages its biological activities for survival and reproduction. At the level of transcription, specific transcription factors (TFs) bind DNA in order to activate or repress the expression of a gene. MiRNAs repress gene expression post-transcriptionally by interacting with complementary sequences located in the 3′UTR of their target mRNAs [7]. For example, co-expressed genes and genes encoding interacting proteins tend to be regulated by common TFs [8]. Although synthetic genetic interactions mostly occur between homologous genes, large gene families have been identified that complicate interactions between some important genes [9]. Genes encoding TFs that control miRNA expression have a higher chance to be post-transcriptionally repressed by the miRNA. Furthermore, genes co-regulated by miRNAs are less functionally linked than genes co-regulated by TFs. Therefore, different types of molecular interactions provide additional insights and new horizons of valuable information about gene regulation and cell function, which indicates the need and comprehensive scientific attention to integrating data and extracting existing connections and correlations [10, 11].

Gene network analysis (GNA) use computer and bioinformatic data tools to model and simulate genetic regulatory networks. The important aim of GNA is to help life science researchers build a model of a genetic regulatory network using knowledge and information related to regulatory interactions in combination with gene expression [6, 7, 9].

Garlic (*Allium sativum* L.), a diploid (2n = 2x = 16) plant species is an important economic and medicinal plant with more than 5,000 years of planting history on the planet. Pungency of garlic is related to organosulfur components including allicin. Allicin is considered responsible for most of the pharmacological activity of crushed raw garlic cloves [4]. When the tissues of any Allium species are disrupted, these amino acid derivatives are cleaved by the enzyme alliinase (EC 4.4.1.4) into their corresponding sulfenic acids, and volatile sulfur compounds are produced that give the characteristic flavor and bioactivity of the species [5, 6]. Despite its agronomic importance, garlic remains largely a wildl plant, hampering its commercial potential. Garlic is the first species within the Allium genus to be sequenced [11, 12]. According to these sequencing results, garlic experienced a recent occurrence of burst of transposable elements. Alliinase genes and content are thought to have rapidly expanded during the burst of transposable elements which helps explain the evolution of allicin biosynthesis-related genes [13]. Two other genes including sulfite reductase (*SiR*) and superoxide dismutase (*SOD*) are thought related to sulfate assimilation pathway. However, whether it is involved in stress response in crops is largely unknown. These genes are therefore potential candidates of the alliin biosynthesis pathway.

Pungent and weak odor in garlic has its own advantages depending on the purpose of garlic consumption. Certainly, food processing industries as well as spice uses require the maximum aroma and spiciness of garlic, if it is consumed fresh and raw, a little spiciness of garlic can be desirable. Genetic manipulation or environmental and agricultural interventions to achieve the various goals above will require detailed knowledge on the network of related genes involved in the biosynthesis of spiciness in garlic and onion.

## Materials and methods

### Plant materials

**Fig. 1:**
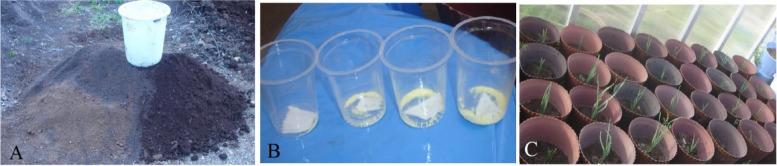
Preparation of uniform agricultural soil including two parts of garden soil, two parts of sand and one part of rotted manure (**A**). Different concentrations of elemental sulfur (**B**). The growth of garlic clones in different concentrations of sulfur (**C**)

The academic permission for collect and research on medicinal plants was obtained from Head of Biotechnology, Department Research Institute of Modern Biological Techniques, University of Zanjan, Zanjan, Iran. The study complies with all relevant guidelines. Three different garlic clones (with high, medium and low odor) (12) that had been selected based on pungency spectrophotometric method [12] were cultured in pots (Fig. 1) containing different concentrations of sulfur (0, 6, 12, 18, 24, 60 g.). *Thiobacillus* bacteria were added to each pot containing 1 kg of soil. The pots were given a month to undergo for oxidation process of sulfur and the dissolved sulfur in the soil. After one month, 3 mentioned clones were planted in three replicates, in depths of 5 cm. After three months of cultivation and at the end of the growing season, the plants were harvested and the garlic cloves harvested were used for RNA extraction and gene expression studies.

### Gene expression analysis

RNAs were extracted based on Ribo Spin Plant Kit, which prepared of GeneAll Oguem-dong, Songpa-gu, Seoul, Korea. The cDNAs were synthesized using Thermo kit (GeneAll Oguem-dong, Songpa-gu, Seoul, Korea) accordingly [14]. Expression of three pungency related genes including *All*, *SiR* and *SOD* (Table 1) were evaluated under sulfur concentrations (0, 6, 12, 18, 24 and 60 g) using q-RT-PCR. The characteristics of studied primers were shown in Table 1 [12].

**Table 1:** Designed primers were used in q-RT-PCR for relative expression analysis of *All*, *SiR* and *SOD* genes [12]

Primer name	5'— 3'	5'— 3'
SOD1	GTGAAGGCTGTTGCTGTT	CCTTGGAGACCAATGATACC
SOD2	GGCGACCTAGGAAATGTGAC	ATACCGCATGCAATTCTTCC
SIR1	TGATATTCTCAAAAGGGTGCAA	GGGAAATAAGAATCAGTGGTGA
SIR2	CCTGCTCTGCCTCTATGTCC	CTATTTGGGCCATCACCAAC
ALI1	ATGGTGAAAACGCAGAAAGG	CATTCACACTTCACCCATGC
ALI2	GGCTGTAGCGGCAGTCTACT	TGTCGTAGTTGTACCCAGACG
Actin	TCCTAACCGAGCGAGGCTTCAT	GGAAAAGCACTTCTGGGCACC

### Gene network analysis

To distinguish and select significant pathways in sulfur metabolism, RESNET Plant database of Pathway Studio software v.10 (Elsevier), and other relative data such as chemical reactions, TFs, miRNAs, enzymes, and small molecules were extracted. This database includes aliases for genes in the model plants and the other plants including tobacco, and tomato. To predict interaction between genes and sulfur molecule, various statistical tests such as, Fisher’s Exact Test were used [15, 16]. To make statistical network based on sulfur and candidate genes, union selected, physical and direct interaction algorithms were used.

### Promoter analysis

Promoter analysis can provide valuable information about underlying regulatory mechanism and function of genes in response to lots of signals [17, 18]. The sequence of *SIR* (AT5G04590) of *Arabidopsis* (as a model plant) was downloaded from the NCBI database (www.ncbi.nlm.nih.gov). 1500 Kb upstream (from the start codon) was extracted as promoter sequences from Ensembl through the BioMart tool (http://plants.ensembl.org/biomart). To find the transcription factors and their binding sites across the promoter region Plantpan v.2 database was used (http://plantpan2.itps.ncku.edu.tw/promoter.php) [19].

## Result and discussion

**Fig. 2:**
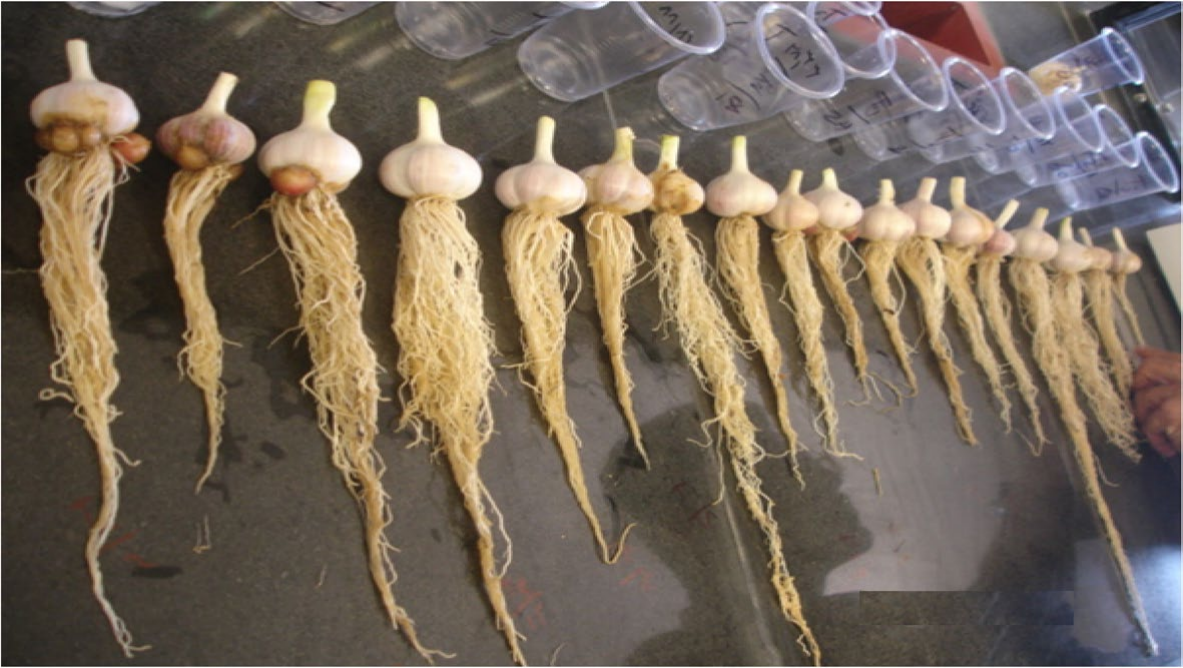
Studied garlic clones under sulfur treatments that were used for pungency related network analysis

**Fig. 3:**
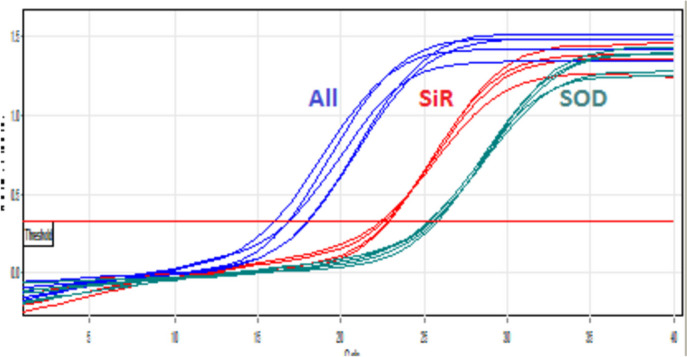
The amplification curve *of* *ALL, SiR and SOD* genes in different concentrations of sulfur

Some of used garlic clones for this research were present in Fig. 2. The sequence and priority of the simultaneous expression of the three candidate genes compared to each other is shown in Fig. 3. Gene expression analysis revealed the highest expression in *All* and *SiR* genes respectively.

**Fig. 4:**
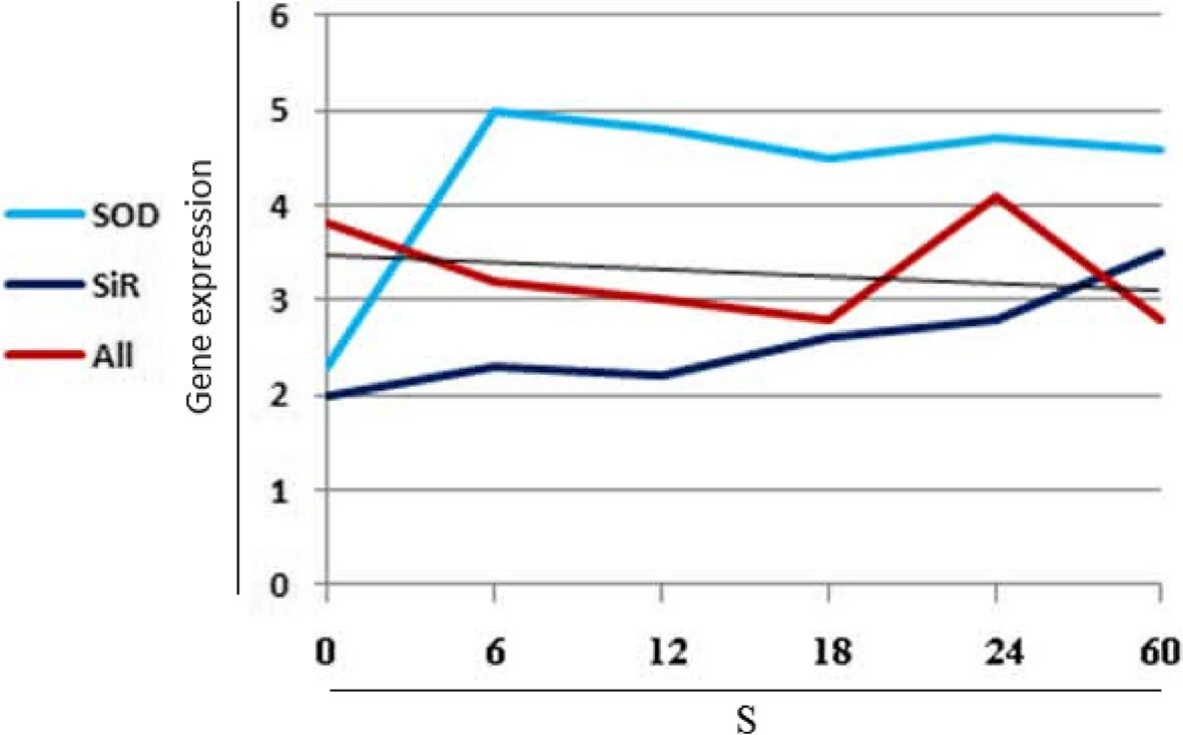
Expression changes of three genes under sulfur concentrations (0, 6, 12, 24 and 60 g/ per experimental unit: pot) using real-time quantitative PCR (RT-qPCR) analysis. Unlike the other two genes, the allinase (*All*) gene faces a decrease in gene expression up to the concentration of 18 sulfur and it faces sudden changes in the concentration of 18 to 24 and the level of gene expression increases and then starts a downward trend. The *SOD* gene is associated with an upward increase in the initial concentrations of sulfur, and then there is a very slow decrease. But gene *SiR* shows a continuous and slow rate of increasing expression

Allinase gene is the first that was observed in this study in the first place in the amount and speed of expression. The next gene is the *SiR* and the third gene in terms of the speed of gene expression is *SOD*. This graph specifically shows the order and priority of expression of the three mentioned genes at the same time. Because the focus of the research is on the study of the in silico gene network involved in the absorption and transport of sulfur in the garlic plant, therefore, we have not provided the molecular data of gene expression changes here, and we only wanted to provide the necessary primary documentation give to the readers including experimental and field works (Fig. 1), providing the primers (Table 1) and the expression order of the three studied genes (Fig. 3). However, the changes in the expression of the three studied genes are shown in Fig. 4.

**Fig. 5:**
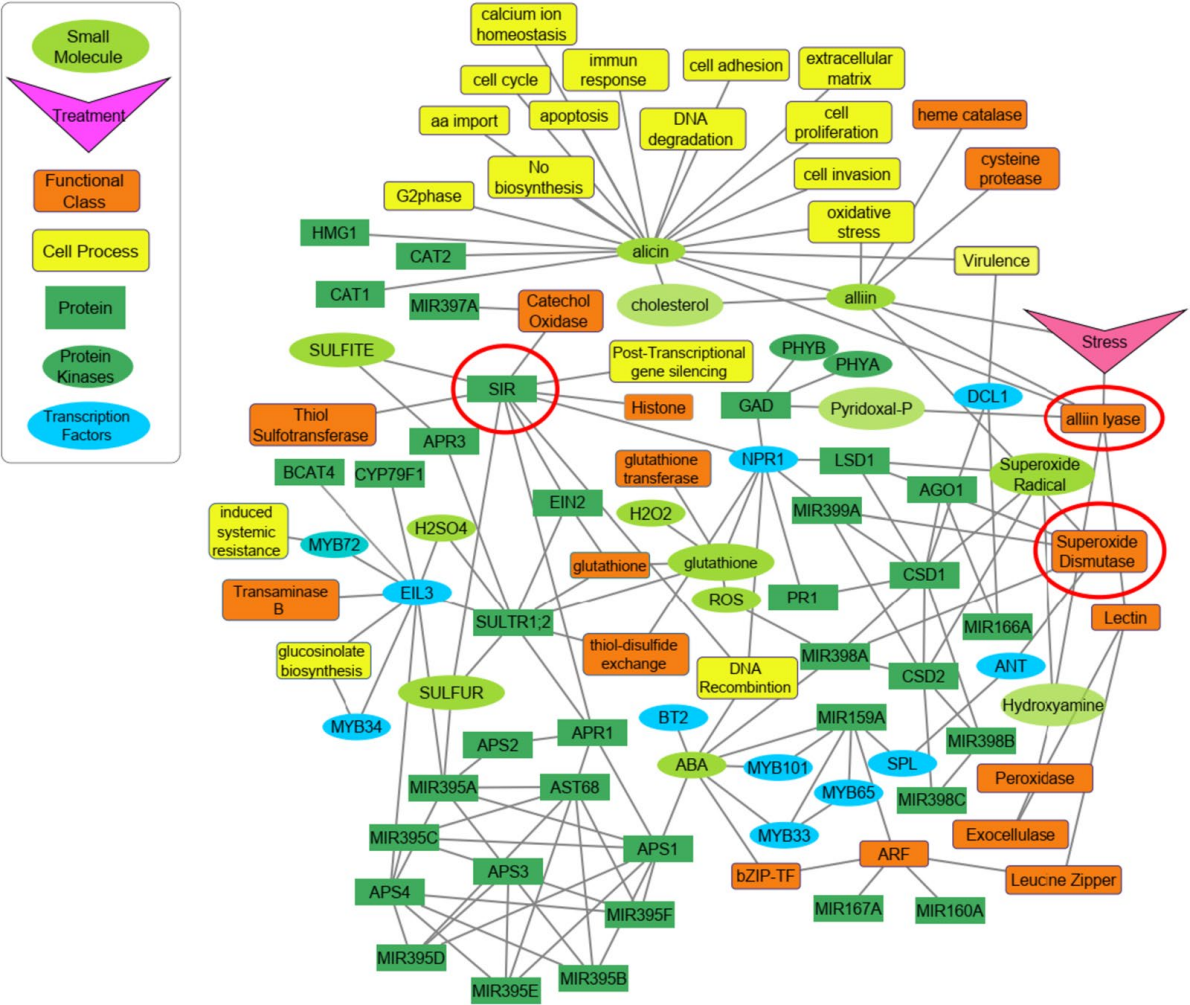
Sub-network of candidate genes (those with red circles) between sulfur molecules in *Allium sativum* L. Network constructed using Pathway Studio version 10

**Fig. 6:**
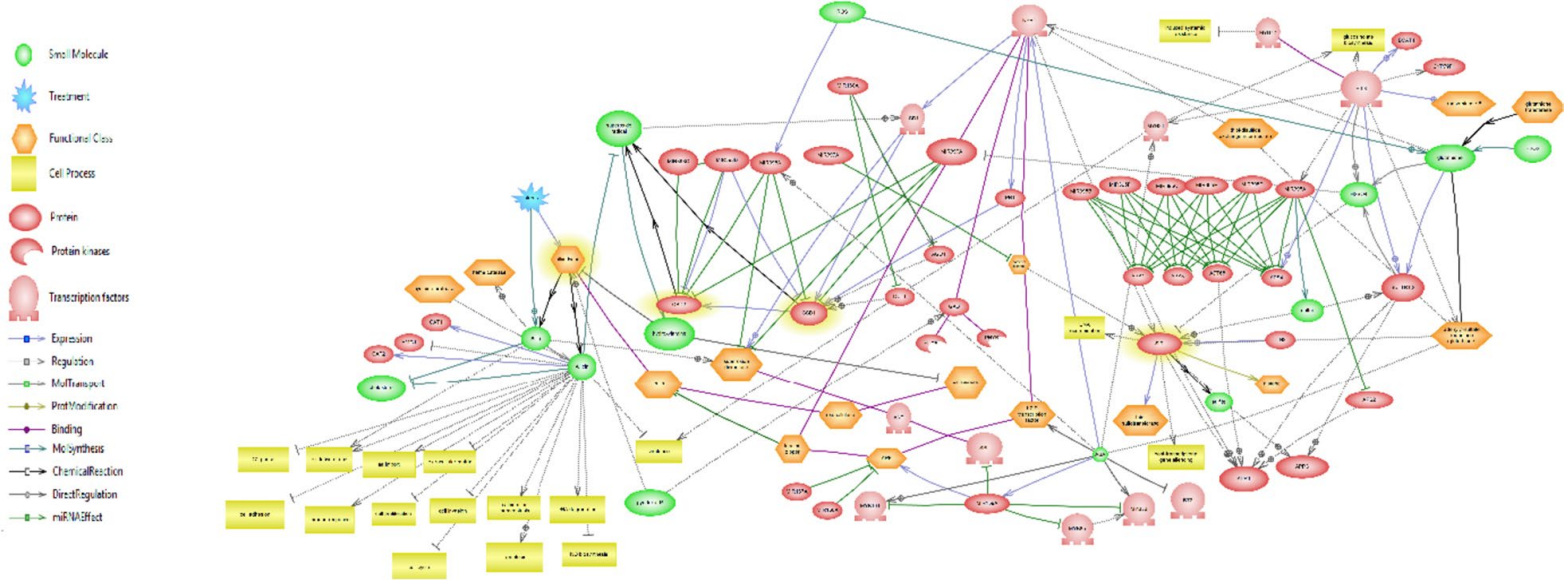
Sub-network of candidate genes between sulfur molecule in *Allium sativum* L. and interaction genes, miRNAs, TFs, stress, cell process, and functional class. Network constructed using Pathway Studio version 10. Genes being analyzed are highlighted in yellow

A gene network was constructed to investigate various mechanisms stimulation of *A. sativum* L. to cope with imposed stress (Fig. 5). To closer inspect, complicated network, including TF-TF, miRNA-TF, and miRNA-TF-gene, was split into sub-network. Figure 6, complicated sub- network, exhibited plenty of routes between stress response and sulfate assimilation pathway.

GO analysis is a strong approach in understanding the molecular mechanisms underpinning developmental and environmental processes and offer a reliable tool for GO gene selection [20]. Integration network revealed the genes are involved in various biological processes such as, RNA splicing, stress response, gene silencing by miRNAs, and epigenetic.

### Ethylene-insensitive3-like 3, EIL3 (SLIM1) key regulator in sulfur assimilation and garlic pungency

*EIL3* is considered as central hub in sulfur response and metabolism [21]. Network analysis showed *EIL3* controls lots of genes involving not only in sulfur assimilation such as, *miR395*, *SULTR1;2*, and *APS4*, but also glucosinolate biosynthesis process such as *MYB34* [22, 23]. Glucosinolates, secondary metabolites, has roles in plant defense and inducers of anticarcinogenic in human [24]. In addition, Allicin has a huge number of activities such as, immune response, inhibiting the proliferation of tumor cells and induced apoptosis in gastric epithelial, breast cancer cells (Fig. 5). Since abundance of *Allinase* was high under normal condition, and plant needs to assimilate sulfur, it could be concluded that the sulfur uptake and assimilation, the intensity of pungency and drug metabolic in garlic might be controlled by *EIL3*.

### *Alliinase* interplay between oxidase and metabolic pathways

Even though *Alliinase* did not display any connectivity with other stress response genes, it showed binding relation with lectin functional class, as a result of which connected to leucine zipper, exocellulase, peroxidase and ARF functional class indirectly (Fig. 5). *ARFs* (Auxin Response Factors) families involved in auxin signaling are regulated by *miR167* and *miR160* under abiotic stress [15, 25] Maruyama-Nakashita [20] reported expression of *SULTR1;1* was induced by ARFs and Aux/IAA proteins under sulfur deficiency.

Moreover, Because of having two domains, EGF_alliinase (PF04863) and Alliinase_C (PF04864)*,* it is presumed that *Alliinase* might be a part of primitive plant defense response. Therefore, this gene is speculated to be a significant linker between secondary metabolic and abiotic stress tolerance pathways.

### Nonexpresser of PR genes 1 (NPR1) prior regulator in response to sulfur stress

Network analysis of *SIR* showed interaction with histone functional class, and post-transcriptional gene silencing [26, 27], and its expression is controlled by *NPR1*, central key in response to salt and oxidative stress tolerance in *Arabidopsis* [28].

Sub-network showed the expression of *NPR1* is affected by glutathione, redox signaling molecule in defense response [29, 30] and interacts with *leucine zipper* and *bZIP* transcription factor functional classes. Moreover, *CSDs* are regulated by *miR398*, *DCL1*, involved in various biological processes such as RNA interferences, gene silencing by miRNAs and production of miRNAs, and *LSD,* is regulated by *NPR1*; as a consequence, *NPR1* regulate them indirectly (Fig. 6).

Since *SIR* induced earlier than *CSDs* during sulfur supply conditions, *NPR1* might be considered as a pivotal factor in response to stress. In addition, promoter analysis of *SIR* revealed that most of transcription factors belong to *bHLH* and *bZIP* family such as *REV*, *NF-YA10*, *NF-YB1*, and *NF-YA2*, which produce the high number of alternative splicing variants in barely. Alternative splicing might act as regulatory link between miRNAs and stress response [31]. Therefore, *SIR* might be considered as a key mediator gene signaling pathway in response to stress in garlic.

## Conclusion

For the modern biologist, there are numerous computational strategies that can be employed to assay gene expression. Many of these are based on utilizing collections of expressed sequence tags (ESTs), unique segments of cDNA with base sequences identical to at least part of the coding region of a gene [20, 32]. Gene expression network reconstruction and analysis are starting to be widely used to characterize and predict biosystem behavior, giving rise to a new branch of biological knowledge, ‘network genomics’ [33]. Until recently, such analyses have been limited to one level of manifestation of the genetic information, i.e. transcript networks [34] or metabolic networks (https://doi.org/10.1371/journal.pone.0058759). However, changes in transcript levels are transferred to changes in metabolite levels and thereby to physiological endpoints via adaptations of physiology and homeostasis [35]. Complicated sub- network, exhibited plenty of routes between stress response and sulfate assimilation pathway. Even though *Alliinase* (*alliin lyase*) did not display any connectivity with other stress response genes, it showed binding relation with lectin functional class, as a result of which connected to leucine zipper, exocellulase, peroxidase and ARF functional class indirectly. Integration network of these genes revealed they are involved in various biological processes such as, RNA splicing, stress response, and gene silencing by miRNAs. In this research, the order and priority of simultaneous expression of three important genes including *All*, *SiR* and *SOD* were measured on some garlic ecotypes of Iran. Also, based on total information resulted of simultaneous amplification curve of *ALL, SiR* and *SOD* genes, in silico gene network related to these three key genes were presented. The findings of this research can be used in further research on the garlic metabolic engineering, garlic stress related genes, and also reducing or enhancing the activity of the responsible genes for garlic pungency and health beneficial. This pungency related network analyzing that is first report on garlic pungency, let us total view for distribution and function of related genes.

## Data Availability

Data sharing is not applicable to this article as no datasets were generated or analyzed during the current study.
